# Michael-type addition of azoles of broad-scale acidity to methyl acrylate

**DOI:** 10.3762/bjoc.7.24

**Published:** 2011-02-08

**Authors:** Sławomir Boncel, Kinga Saletra, Barbara Hefczyc, Krzysztof Z Walczak

**Affiliations:** 1 Silesian University of Technology, Department of Organic Chemistry, Bioorganic Chemistry and Biotechnology, Krzywoustego 4, 44-100 Gliwice, Poland, Tel.: +48 32 237 1792, Fax: +48 32 237 2094; 2 Silesian University of Technology, Department of Chemical Organic Technology and Petrochemistry, Krzywoustego 4, 44-100 Gliwice, Poland, tel.: +48 32 237 10 32, fax: +48 32 237 10 32

**Keywords:** imidazole derivatives, methyl acrylate, Michael-type addition, pyrazole derivatives, 1,2,4-triazole derivatives

## Abstract

An optimisation of Michael-type addition of azole derivatives of broad-scale acidity – ranging from 5.20 to 15.00 p*K*_a_ units – namely 4-nitropyrazole, 3,5-dimethyl-4-nitropyrazole, 4(5)-nitroimidazole, 4,5-diphenylimidazole, 4,5-dicyanoimidazole, 2-methyl-4(5)-nitroimidazole, 5(4)-bromo-2-methyl-4(5)-nitroimidazole and 3-nitro-1,2,4-triazole to methyl acrylate as an acceptor was carried out. The optimisation process involved the use of an appropriate basic catalyst (DBU, DIPEA, NaOH, NaH, TEDA), a donor/base/acceptor ratio and the reaction temperature. The reactions were performed in DMF as solvent. Target Michael adducts were obtained in medium to excellent yields. Importantly, for imidazole and 1,2,4-triazole derivatives, no corresponding regioisomers were obtained.

## Introduction

**Figure 1:**
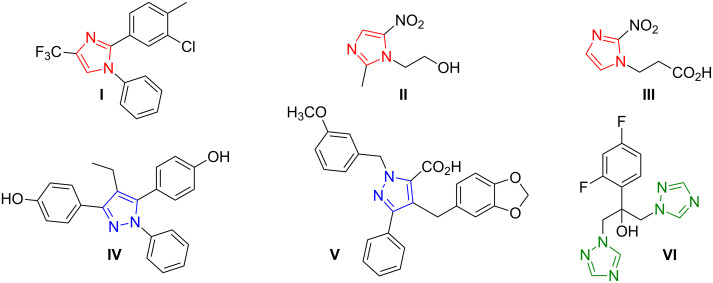
Examples of azole derivatives as important therapeutic agents.

Derivatives of azoles are important biologically active compounds. Many, especially those containing imidazole, pyrazole and 1,2,4-triazole moieties, constitute building blocks for a variety of therapeutic agents (Figure 1).

1,2-Arylimidazole derivatives, e.g., 2-(3-chloro-4-methylphenyl)-1-phenyl-4-(trifluoro-methyl)imidazole (**I**) are selective cyclooxygenase (COX-2) inhibitors [1]. Metronidazole (2-(2-methyl-5-nitroimidazol-1-yl)ethanol) is an antibiotic and antiprotozoal agent whose uses include, among others, the treatment of anaerobic bacterial infections [2]. Complexes of Co(II) and Cu(II) with 3-(2-nitroimidazol-1-yl)propanoic acid (**III**) are radiosensitisers in cancer therapy [3]. 4,4'-(4-Ethyl-1-phenylpyrazole-3,5-diyl)diphenol (**IV**) and its derivatives are known modulators of receptors regulating the secretion of estrogens crucial in the pathogenesis of breast cancer [4]. 4-(Benzo[*d*][1,3]dioxol-5-ylmethyl)-1-(3-methoxybenzyl)-3-phenyl-pyrazole-5-carboxylic acid (**V**) is an antagonist of endothelial receptors and is used as a clinical regulator of the cardiac cycle [5]. Fluconazole (**VI**) 2-(2,4-difluorophenyl)-1,3-bis(1,2,4-triazol-1-yl)propan-2-ol is an antifungal drug used in the treatment and prevention of superficial and systemic fungal infections [6].

The base-catalysed Michael-type addition reaction of various azoles to α,β-unsaturated carbonyl and nitro derivatives has been frequently exploited in the synthesis of precursors of biologically active compounds [3,7–15]. This reaction, although structurally restricted to α,β-unsaturated carbonyl compounds and their analogues, is advantageous due to its higher regioselectivity compared to alkylation reactions using alkyl halides [16–19], alkyl sulfates [20] or oxiranes [18] under basic conditions, or alcohols over zeolites [21]. Moreover, it is usually described as a green-chemistry with an effective synthetic protocol and a simple work-up. In the Michael-type additions of azole N–H acids, numerous catalysts such as KF/Al_2_O_3_ [8], CeCl_3_∙7 H_2_O/NaI [9], NaH or ZnCl_2_ [10], K_2_CO_3_ [11], anhydrous K_3_PO_4_ [12], (NH_4_)_2_Ce(NO_3_)_6_ (CAN) [22] or ionic liquids, e.g., Cu(acac)_2_ immobilised in [bmim][BF_4_] [15] have been used. Other reaction conditions including enzymatic catalysis (*Bacillus subtilis*) [13], zinc-active-site acylases from *Escherichia coli* and *Aspergillus oryzae* [23] as well as ultrasonic irradiation in the presence of montmorillonite [14] have also been reported. In addition, there has been several recent reports on the synthesis of Michael-type adducts of azoles by the use of microwave irradiation [7,24]. The optimisation of aza-Michael reactions with *N*-methylimidazole as the base catalyst was published in 2007 by Liu et al [25]. However, when this catalyst was used with azoles of narrower-scale acidity (p*K*_a_ range from 8.93 to 15.1) including imidazole and its 2- and 4(5)-methyl, 4(5)-nitro-, 2-methyl-4(5)-nitro- derivatives as well as with 1,2,4-triazole, there was no evidence for a regioselective course of the reactions. Furthermore, extremely high acidic azoles, which are substrates for many important drugs, have seldomly been considered as aza-Michael donors due to their poor nucleophilicity and the possible tendency of adducts to undergo retro-Michael reactions [26].

Here, we present Michael-type addition of azoles, inter alia imidazole and 1,2,4-triazole derivatives of high acidity (p*K*_a_ ≤ 6.05), to methyl acrylate under optimised conditions (temperature, type of base and donor/base/acceptor ratio) as a regioselective, efficient and useful reaction for the formation of adducts that can be utilised in the synthesis of more complex systems including intermediates for the pharmaceutical industry[27].

## Results and Discussion

**Scheme 1:**
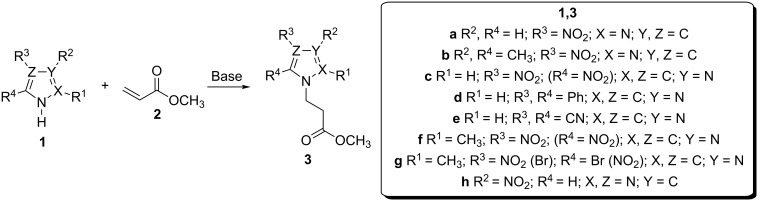
Michael-type addition of azoles of broad-scale acidity **1a–h** to methyl acrylate (**2**) under basic conditions.

4-Nitropyrazole (**1a**), 3,5-dimethyl-4-nitropyrazole (**1b**), 4(5)-nitroimidazole (**1c**), 4,5-diphenylimidazole (**1d**), 4,5-dicyanoimidazole (**1e**), 2-methyl-4(5)-nitroimidazole (**1f**), 4(5)-bromo-2-methyl-5(4)-nitroimidazole (**1g**) and 3-nitro-1,2,4-triazole (**1h**) were used as Michael donors. The azoles derivatives (**1a**–**h**) were subjected to the reaction with methyl acrylate (**2**) (Michael acceptor) in the presence of an appropriate base, namely DBU (p*K*_a_ = 12 [28]), diisopropylethylamine (DIPEA, Hünig’s base, p*K*_a_ = 10.75 [29]), NaOH (p*K*_a_ = 15.7 [30]), NaH (p*K*_a_ = 37 [29]) or TEDA (p*K*_a_ = 8.82 [31]) in polar aprotic (DMF) or protic (MeOH) solvent (Scheme 1, Table 1). Although the above p*K*_a_ values were determined in water, their relative basicity is usually in good agreement in other polar solvents, e.g., there is a linear relationship between p*K*_a_ values in water and DMF for the set of *N*-centred organic bases expressed by the equation: p*K*_a_ (DMF) = 0.9463 p*K*_a_ (water) + 1.4154 [32–33]. This semi-empirical relationship enables an initial selection of a base required for a sufficient level of deprotonation of N–H azole acids.

**Table 1:** Optimised conditions of Michael-type addition of azoles of broad-scale acidity **1a–h** to methyl acrylate **(2)**, physical properties and yields of the products **3a–h**.

**1**	p*K*_a_ (H_2_O)	Conditions					**3**

		A/D	Base	Base / A	*T* [°C]	*t* [h]	Yield [%]

**a**	9.67 [34]	1:1	DBU	1.0	20	1	31
		2:1	DIPEA	1.0	20	5	97

**b**	15.00 [35]	1:1	DBU	1.0	20	1	54
		2:1	DIPEA	1.0	20	24	98

**c**	8.93 [36]	1:1	DBU	1.0	20	2	30
		1.1:1	DIPEA	1.0	20	12	98

**d**	5.70^a^ [37]	4:1	DIPEA	1.0	20	192	0
		4:1	DIPEA	1.0	80	48	0
		1:1	w/o^b^	—	60	168	0
		4:1	NaOH^b,c^	0.02	65	24	8
		2:1	NaH	1.0	60	72	60

**e**	5.20 [38]	2:1	DIPEA	1.0	20	192	0
		1:1	DIPEA	1.0	60	120	0
		1:1	DBU	1.0	60	120	0
		2:1	NaOH^c^	1.0	65	24	25
		1:1	NaH^b,d^	2.5	60	144	0
		1.5:1	NaH^b,d^	1.2	60	144	0
		1:2	NaH^b,d^	1.2	60	144	14
		1:2	NaH^b,d^	1.2	60	168	62

**f**	9.64 [39]	1:1	DIPEA	1.0	20	120	97

**g**	—	3:1	DIPEA	1.0	60	96	22^e^
		2:1	DBU	1.0	20	72	0
		1:1	TEDA	1.0	20	120	0
		2:1	NaOH^c^	0.02	65	48	13
		1:2	NaH^b,d^	1.2	60	72	55

**h**	6.05 [18]	2:1	DIPEA	1.0	20	114	80

^a^30 mol % DMSO aqueous solution at 30 °C, ^b^Reaction performed under an inert (N_2_) atmosphere, ^c^reaction performed in MeOH, ^d^80% solution of NaH in mineral oil was used; ^e^total yield, a regioisomer **4g** was obtained in 10% yield (^1^H NMR); A/D = acceptor to donor molar ratio.

**Scheme 2:**
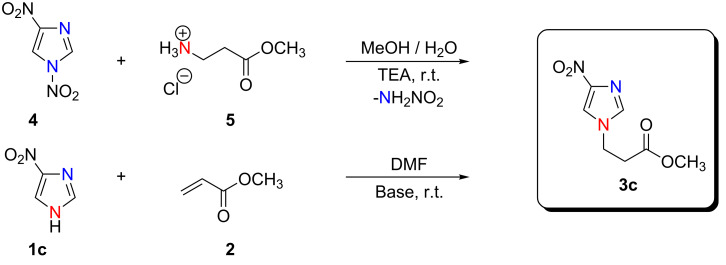
Chemical evidence for a regioselective Michael-type addition of 4(5)-nitroimidazole (**1c**) to methyl acrylate (**2**) based on the ANRORC reaction of 1,4-dinitroimidazole (**4**) with β-alanine methyl ester hydrochloride (**5**).

**1a** and **1b** as weak N–H acids required an equimolar amount of a strong base (DBU) and reacted with **2** in stoichiometric proportions to give the appropriate products **3a** and **3b** in high purity but in low/medium yields. The presence of the unreacted substrates **1a**, **1b** in the post-reaction mixtures points to the tendency of the adducts to undergo the reverse reaction – a retro-Michael reaction – in equilibrium with Michael-type addition. By contrast, DIPEA, with a lower p*K*_a_ gave the same adducts **3a** and **3b** in excellent yields. For **1b** (the azole with the lowest acidity) a similar yield to **1a** was achieved only after a significantly prolonged reaction time due to its lower concentration of the anionic form in the reaction mixture. For **1c**, analogous behaviour was observed when treated with **2** in the presence of DBU. Here again, a use of DIPEA, instead of DBU, in a slight molar excess with respect to the azole furnished **3c** in excellent yield. These conditions were also adequate in the reactions of **1f** and **1h** with **2** and gave the corresponding products **3f** and **3h** respectively, in excellent yields. No regioisomers were obtained for these *C*-nitro azole derivatives. The structure of **3c** was established by an independent synthesis from 1,4-dinitroimidazole (**4**) and β-alanine methyl ester hydrochloride (**5**). This latter reaction proceeds via an ANRORC mechanism (Addition of Nucleophile, Ring Opening, Ring Closure) [40–41] (Scheme 2). Product **3c** obtained by the two independent routes, possessed identical physicochemical and spectral properties. The observed difference in yields, 98% for Michael-type addition and 60% for ANRORC, arises from some unidentified side reactions.

In the case of the reaction of **1d**, an imidazole derivative of acidity three orders of magnitude higher compared to **1c**, no reaction was observed in the presence of an equimolar amount of DIPEA and even with a four-fold molar excess of the acceptor at ambient or elevated temperature. We then carried out an additional trial in the absence of a deprotonating agent at elevated temperature on the assumption that due to the increased acidity of **1d**, a small concentration of the anionic form would be sufficient for completion of the reaction. However, gain no reaction was observed. On the other hand, the use of a catalytic amount of NaOH at elevated temperature furnished only a small amount of the expected product **3d**. However, when **1d** was treated with a double molar excess of the acceptor in the presence of NaH at elevated temperature, the expected product was isolated in good yield (60%). The reaction of the most acidic imidazole derivative within the group **1e** was more complicated to optimise. DBU, DIPEA and NaOH all appeared as ineffective basic catalysts and only when a double molar excess of the donor was used in the presence of NaH was a satisfactory yield of **3e** obtained. Similarly, for **1g** only the former conditions gave **3g** in good yield (62%). Interestingly, a regioisomeric product of the addition, i.e., methyl 3-(4-bromo-2-methyl-5-nitroimidazol-1-yl)propanoate (**4g**) was not obtained when NaH was used as a catalyst. However, when DIPEA was used as a base, this regioisomer was obtained in ca. 10% yield, as determined by ^1^H NMR spectroscopy. The structures of all the products were established by means of ^1^H and ^13^C NMR spectroscopy.

It is worth noting that in none of the reactions polymerisation or hydrolysis of methyl acrylate was observed. Nevertheless, decreased yields for azole adducts when DBU or NaH were used as catalysts at elevated temperature could originate from a reversibility of the Michael reaction since unreacted azoles were isolated. This observation is in a good agreement with our recent findings for Michael-type addition of uracils to acrylic acceptors [27,42], where a a strongly basic catalyst (DBU) enabled control of regioselectivity via a retro-Michael reaction.

## Conclusion

We have developed and optimised a simple and effective synthetic protocol for Michael-type addition of azoles of broad-scale acidity including pyrazole, imidazole and 1,2,4-triazole derivatives. Importantly, for 4(5)-nitroimidazole, 2-methyl-4(5)-nitroimidazole, 4(5)-bromo-2-methyl-5(4)-nitroimidazole and 3-nitro-1,2,4-triazole no regioisomers were obtained. The adducts constitute biologically active model compounds themselves and which, after acidic hydrolysis [43], can be anchored to drug delivery systems, including, e.g., chemically modified carbon nanotubes, via amide bonds.

## Supporting Information

File 1 Experimental part.
