# Supplementary material for: Michael-type addition of azoles of broad-scale acidity to methyl acrylate
Source: Beilstein J Org Chem. 2011 Feb 8;7:173–8. doi: 10.3762/bjoc.7.24 (PMC3063008; doi:10.3762/bjoc.7.24)
Supplement: File 1 — Experimental part. [file Beilstein_J_Org_Chem-07-173-s001.pdf]

Supporting Information

for

Michael-type addition of azoles of broad-scale acidity to methyl acrylate

Sławomir Boncel*[1], Kinga Saletra[1], Barbara Hefczyc[2], Krzysztof Z. Walczak*[1]

Address: [1]Silesian University of Technology, Department of Organic Chemistry, Bioorganic Chemistry and Biotechnology, Krzywoustego 4, 44-100 Gliwice, Poland, Tel.: +48 32 237 1792, Fax: +48 32 237 2094 and [2]Silesian University of Technology, Department of Chemical Organic Technology and Petrochemistry, Krzywoustego 4, 44-100 Gliwice, Poland, tel.: +48 32 237 10 32, fax: +48 32 237 10 32

E-mail: Sławomir Boncel* - slawomir.boncel@polsl.pl, Kinga Saletra - kingasaletra@gmail.com, Barbara Hefczyc - barbara.hefczyc@polsl.pl, Krzysztof Z. Walczak* - krzysztof.walczak@polsl.pl

* Corresponding author

Experimental part

General remarks

NMR spectra were recorded at 300 MHz for ^{1}H NMR and 75.5 MHz for ^{13}C NMR on a Varian Inova 300 MHz; *δ* values are in parts per million (ppm) relative to tetramethylsilane (TMS) as an internal standard. Elemental analyses were obtained using a Perkin-Elmer 240C apparatus. Mass spectra were recorded on GC/MS Agilent Technologies 7890A/5975C. All used reagents were purchased from Aldrich or Alfa Aesar. TLC $60F_{254}$ plates and silica gel 60 (0.040–0.063 mm) were purchased from Merck. Melting points were measured at Boetius apparatus and are uncorrected. DMF was distilled prior to use and stored over molecular sieves 4 Å.

Michael adducts (3a–h, 4g) – general procedure

A solution of azole **1a–h** (1 mmol) in an appropriate solvent (6 mL) was magnetically stirred and maintained (if necessary) under an atmosphere of nitrogen. The deprotonating agent and methyl acrylate (**2**) were added in the ratio given in Table 1. Optional heating of the reaction mixture was performed using an oil bath. The progress of the reactions was monitored by TLC ($MeOH/CHCl_3$). After consumption of the limiting reactant, the reactions were quenched with 35% HCl_{aq}. From the post-reaction mixtures volatiles were removed under reduced pressure using a rotary evaporator. The residues were isolated and purified by silica gel column chromatography with 10% $MeOH/CHCl_3$ (v/v) as an eluting system.

Methyl 3-(4-nitro-1*H*-pyrazol-1-yl)propanoate (3a)

From 0.113 g (1 mmol) **1a**, 0.062 g of **3a** was obtained as a yellow oil, yield 97%. ^{1}H NMR: (DMSO-d_6), δ (ppm): 2.97 (t, 2H, *J* = 6.6 Hz; H-2); 3.61 (s, 3H, OMe); 4.44 (t, 2H, *J* = 6.6 Hz, C-2); 8.25 (s, 1H, C-5’); 8.90 (s, 1H, C-3’). ^{13}C NMR: (DMSO-d_6), δ (ppm): 33.1 (C-2); 48.0 (C-3); 51.6 (OMe); 130.8 (C-5’); 134.8 (C-4’); 135.6 (C-3’); 170.7 (C-1). $C_7H_9N_3O_4$ (199.16). MS, *m/z*: 59 (33%), 68 (20%), 79 (61%), 85 (50%), 121 (49%), 139 (54%), 140 (56%), 152 (23%), 168 (37%), 182 (M^+, 100%), 199 (45%).

Methyl 3-(3,5-dimethyl-4-nitro-1*H*-pyrazol-1-yl)propanoate (3b)

From 0.141 g (1 mmol) **1b**, 0.123 g of **3b** was obtained as a yellowish oil, yield 98%. ^{1}H NMR: (DMSO-d_6), δ (ppm): 2.38 (s, 3H, CH_3 imidazole); 2.62 (s, 3H, CH_3 imidazole); 2.91 (t, 2H, J = 6.6 Hz, H-3); 3.61 (s, 3H, OMe); 4.30 (t, 2H, J = 6.6 Hz, H-2). ^{13}C NMR: (DMSO-d_6), δ (ppm): 11.0 (CH$_3$-C-5'); 13.9 (CH$_3$-C-3'); 32.9 (C-2); 44.4 (C-3); 51.6 (OMe); 130.1 (C-4'); 141.1 (C-5'); 145.1 (C-3'); 170.9 (C-1). Calcd. for $C_9H_{13}N_3O_4$: C, 47.57; H, 5.77; N, 18.49; found C, 47.81; H, 5.85; N, 18.19.

Methyl 3-(4-nitro-1*H*-imidazol-1-yl)propanoate (3c)

Route 1, via the Michael-type addition

From 0.110 g (1 mmol) **1c**, 0.196 g of **3c** was obtained as a yellow powder, yield 98%, m.p. 103–104 °C). ^{1}H NMR: ($CDCl_3$), δ (ppm): 2.89 (t, 2H, J = 6,2 Hz, H-3); 3.73 (s, 3H, OMe); 4.39 (t, 2H, J = 6.2 Hz, H-2); 7.55 (s, 1H, H-5'); 7.88 (s, 1H, H-2'). ^{13}C NMR: ($CDCl_3$), δ (ppm): 35.1 (C-2); 43.7 (C-3); 52.5 (OMe); 119.7 (C-4'); 136.6 (C-5'); 148.2 (C-2'); 170.6 (C-1). $C_7H_9N_3O_4$ (199.16). MS, *m/z*: 59 (47%), 87 (29%), 139 (24%), 140 (100%), 199 (M^+, 46%).

Route 2, via the ANRORC reaction

To 0.16 g (1 mmol) 1,4-dinitroimidazole (**4**) dissolved in methanol (6 mL), 0.14 g (1 mmol) β-alanine methyl ester hydrochloride (**5**) was added. After complete dissolution of the substrates (5 min), TEA was added in order to generate free amine. The light yellow colour of the reaction mixture immediately changed to orange. The reaction course was monitored using TLC (3% MeOH/$CHCl_3$, v/v) and it was complete within 1 h. From the reaction mixture volatiles were removed by distillation under reduced pressure using a rotary evaporator. The solid residue was dissolved in water (5 mL) and was subsequently extracted with ethyl acetate (5 × 5 mL). The organic layers were combined, dried with anhydrous $MgSO_4$ and evaporated to dryness. The crude product was obtained in 60% yield and possessed identical melting point (103–104 °C) and spectral properties (^{1}H and ^{13}C NMR, MS) to the compound obtained via *Route 1*.

Methyl 3-(4,5-diphenyl-1*H*-imidazol-1-yl)propanoate (3d)

From 0.880 g (4 mmol) **1d**, 0.183 g of **3d** was obtained as a yellow powder, yield 60%, m.p. 104–105 °C). ^{1}H NMR: (DMSO-d_6), δ (ppm): 2.64 (t, 2H, *J* = 6.9 Hz H-3); 3.55 (s, 3H, OMe); 4.05 (t, 2H, *J* = 6.9 Hz, H-2); 7.10–7.20 (m, 3H, Ph); 7.34–7.41 (m, 4H, Ph); 7.51–7.53 (m, 3H, Ph); 7,85 (s, 1H, H-2'). ^{13}C NMR: (DMSO-d_6), δ (ppm): 34.3 (C-2); 40.2 (C-3); 51.5 (OMe); 129.2 (C-5'); 125.9; 126.0; 128.0; 128.9; 130.4; 130.7 (Ph); 134.7 (C-2'); 137.3 (C-4'); 170.7 (C-1). $C_{19}H_{18}N_2O_2$ (306.36), MS, *m/z*: 305.1 (33%), 306.1 (100%), 307.1 (21%).

Methyl 3-(4,5-dicyano-1*H*-imidazol-1-yl)propanoate (3e)

From 0.240 g (2 mmol) **1e**, 0.254 g of **3e** was obtained as a yellowish oil, yield 62%. ^{1}H NMR: ($CDCl_3$), δ (ppm): 3.01 (t, 2H, *J* = 6.6 Hz, H-3); 3.62 (s, 3H, OMe); 4.42 (t, 2H, *J* = 6.6 Hz, H-2); 8.37 (s, 1H, H-2'). ^{13}C NMR: ($CDCl_3$), δ (ppm): 33.4 (C-2); 43.1 (C-3); 51.8 (OMe); 108.6 (C-5'); 112.5 (CN); 112.8 (CN); 120.8 (C-2'); 170.5 (C-1). Calcd. for $C_9H_8N_4O_2$: C, 52.94; H, 3.95; N, 27.44; found C, 52.88; H, 3.91; N, 27.20.

Methyl 3-(2-methyl-5-nitro-1*H*-imidazol-1-yl)propanoate (3f)

From 0.127 g (1 mmol) **1f**, 0.207 g of **3f** was obtained as a dark yellow oil, yield 97%. ^{1}H NMR: ($CDCl_3$), δ (ppm): 2.49 (s, 3H, CH_3 imidazole); 2.86 (t, 2H, *J* = 6.3 Hz, H-3); 3,71 (s, 3H, OMe); 4.28 (t, 2H, *J* = 6.3 Hz, H-2); 7.81 (s, 1H, H-5). ^{13}C NMR: ($CDCl_3$), δ (ppm): 13.2 (CH_3); 34.5 (C-2); 42.6 (C-3); 52.6 (OMe); 120.2 (C-4'); 145.2 (C-5'); 146.6 (C-2'); 170.6 (C-1). Calcd. for $C_8H_{11}N_3O_4$ (213.19) C, 45.07; H, 5.20; N, 19.71; found C, 44.88; H, 5.12; N, 19.58.

Methyl 3-(5-bromo-2-methyl-4-nitro-1*H*-imidazol-1-yl) propanoate (3g)

From 0.830 g (4 mmol) **1g**, 0.320 g of **3g** was obtained as a yellow powder, yield 55%, m.p. 105–105.5 °C. ^{1}H NMR: (DMSO-d_6), δ (ppm): 2.47 (s, 3H, CH_3 imidazole); 2.84 (t, 2H, *J* = 7.4 Hz, H-2); 3.63 (s, 3H, OMe); 4.29 (t, 2H, *J* = 7.4 Hz, H-3); ^{13}C NMR: (DMSO-d_6), δ

(ppm): 13.6 (CH_3); 32.7(C-2); 41.7 (C-3); 51.8 (OMe); 95.4 (C-4'); 106.6 (C-2'); 145.8 (C-5'); 170.4 (C-1). ($C_8H_{10}BrN_3O_4$) 292.09. MS, *m/z*: 59 (24%), 87 (25%), 212 (100%), 290.9 (11%) 292.9 (M^+, 11%).

Methyl 3-(4-bromo-2-methyl-5-nitroimidazol-1-yl)propanoate (4g)

Yellow powder, yield 10%. ^{1}H NMR: (DMSO-d_6), δ (ppm): 2.47 (s, 3H, CH_3 imidazole); 2.87 (t, 2H, J = 7.2 Hz, H-2); 3.61 (s, 3H, OMe); 4.51 (t, 2H, J = 7.2 Hz, H-3). $C_8H_{10}BrN_3O_4$: C, 32.90; H, 3.45; Br, 27.36; N, 14.39; found C, 32.90; H, 3.45; Br, 27.36; N, 14.39.

Methyl 3-(3-nitro-1*H*-1,2,4-triazol-1-yl)propanoate (3h)

From 0.114 g (1 mmol) **1h**, 0.160 g of **3h** was obtained as a yellow oil, yield 80%. ^{1}H NMR: (DMSO-d_6), δ (ppm): 3.01 (t, 2H, J = 6.6 Hz, H-3); 3.60 (s, 3H, OMe); 4.57 (t, 2H, J = 6.6 Hz, H-3); 8.85 (s, 1H, C-5'). ^{13}C NMR: (DMSO-d_6), δ (ppm): 32.8 (C-2); 46.2 (C-3); 51.7 (OMe); 146.1 (C-5'); 162.0 (C-3'); 170.7 (C-1). Calcd. for $C_6H_8N_4O_4$: C, 36.00; H, 4.03; N, 27.99; found C, 36.22; H, 3.80; N, 28.33.
